# Developing a Multilevel Polypill Implementation Bundle for Patients With Heart Failure With Reduced Ejection Fraction

**DOI:** 10.1016/j.jacadv.2025.102195

**Published:** 2025-10-08

**Authors:** Justin C. Chen, Colette DeJong, Mansi Agarwal, Amaris M. Hairston, Matthew S. Durstenfeld, Byron Powell, Virginia McKay, Mark D. Huffman, Priscilla Y. Hsue, Anubha Agarwal

**Affiliations:** a Department of Medicine, Washington University in St. Louis School of Medicine, St. Louis, Missouri, USA; b Department of Medicine, Stanford University School of Medicine, Stanford, California, USA; c Institute for Informatics, Data Science, and Biostatistics, Washington University School of Medicine, St. Louis, Missouri, USA; d Department of Medicine, University of Missouri School of Medicine, Columbia, Missouri, USA; e Department of Medicine, University of California-San Francisco, San Francisco, California, USA; f Brown School, Washington University in St. Louis, St. Louis, Missouri, USA; g The George Institute for Global Health, University of New South Wales, Sydney, Australia; h Department of Medicine, David Geffen School of Medicine, Los Angeles, California, USA

**Keywords:** fixed-dose combination pill, heart failure with reduced ejection fraction, implementation mapping, implementation science, polypill

## Abstract

**Background:**

A polypill that contains multiple guideline-directed medical therapies for heart failure with reduced ejection fraction (HFrEF) has been proposed to improve HFrEF outcomes. The factors affecting implementation of a polypill-based strategy for HFrEF are unknown.

**Objectives:**

This study aims to identify determinants that could affect a polypill-based strategy for HFrEF, design a multilevel HFrEF polypill implementation bundle, and illustrate how the bundle could improve outcomes.

**Methods:**

From April to December 2023, we conducted a convergent parallel mixed methods study at Washington University in St. Louis and the University of California-San Francisco to gather patient (N = 9) and provider (N = 22) perspectives on a polypill-based approach to HFrEF care guided by the Consolidated Framework for Implementation Research v2.0. We then used the Consolidated Framework for Implementation Research-Expert Recommendations for Implementing Change Matching Tool to select strategies for a multilevel implementation bundle and mechanism mapping to elucidate how the bundle could improve patient outcomes.

**Results:**

Stakeholder interviews revealed four themes that affect implementation of a polypill-based approach to HFrEF. The *current state of HFrEF care* was the organizing theme, influenced by 3 additional themes: *awareness of new innovations*, *assessing innovation appropriateness*, and *building competency in HFrEF care*. Based on these themes, we developed a multilevel HFrEF polypill implementation bundle with 7 domains and illustrate how the bundle could improve outcomes with a directed acyclic graph.

**Conclusions:**

This study illustrates the development of a multilevel HFrEF polypill implementation bundle that can be further tailored to improve HFrEF care in undertreated populations globally.

Heart failure is a global public health problem affecting over 64 million people worldwide with a 50% 5-year mortality rate.^1^ Although guideline-directed medical therapy (GDMT) for patients with heart failure with reduced ejection fraction (HFrEF) provides an estimated 61% relative reduction in mortality compared to placebo, observational registries in the United States show that only 15% of eligible HFrEF patients receive all four pillars of HFrEF GDMT and patient adherence rates to HFrEF GDMT are persistently low.^2^, ^3^, ^4^, ^5^

A fixed-dose combination pill, or polypill, containing all four HFrEF GDMT medications in one pill has been proposed to improve HFrEF outcomes by promoting patient adherence and guideline-directed HFrEF care.^6^ The SECURE (Secondary Prevention of Cardiovascular Diseases in the Elderly) randomized clinical trial revealed that polypills for secondary prevention of atherosclerotic cardiovascular disease compared to usual care reduce all-cause mortality and cardiovascular events.^7^ Despite these benefits, multiple barriers hinder the widespread adoption, implementation, sustainment, and scale-up of cardiovascular polypills.^8^ Failure to design and adapt such innovations for real-world practice beyond controlled trial settings can delay innovation implementation and increase disparities in patient outcomes.^9^

To address the persistent gap between the discovery of evidence-based interventions and their integration into routine care, the American Heart Association published a scientific statement identifying implementation science as a key area of interest to reduce health care disparities.^10^^,^^11^ While randomized clinical trials are ongoing to evaluate the efficacy and effectiveness of a polypill-based strategy for HFrEF, investigators must also proactively plan how the HFrEF polypill will be implemented to ensure equitable access and adoption.^12^, ^13^, ^14^, ^15^ Building on our previous work identifying key determinants of an HFrEF polypill design and implementation, this study aims to: 1) describe how these determinants interact to influence a polypill-based strategy for HFrEF; 2) develop an HFrEF polypill implementation bundle to address identified determinants; and 3) illustrate how the implementation bundle could improve HFrEF care.^16^

## Methods

From April to December 2023, we conducted a convergent parallel mixed-methods study at multiple academic medical centers, integrating qualitative data from key informant in-depth interviews with quantitative data from stakeholder surveys. We previously reported the methods and results of the study describing key priorities of stakeholders on an HFrEF polypill.^16^ In this report, we focus on insights from key informant in-depth interviews with patients and providers to explore how identified determinants interact to influence implementation of HFrEF polypills. The study was approved by the University of California-San Francisco Institutional Review Board and received an exemption from the Washington University in St. Louis Institutional Review Board.

Prior to interviews, patients completed a survey that included demographic questions and the Beliefs about Medicines Questionnaire (BMQ) (Supplemental Methods).^17^ The BMQ is an 18-item instrument that assesses participants’ perception about medications in general (BMQ-General) and medications related to HFrEF (BMQ-Specific). BMQ-General contains two 4-item subscales with scores ranging from 4 to 20. Higher BMQ-General scores indicate stronger beliefs that medications cause harm (*General-Harm)* or are overused (*General-Overuse)*. BMQ-Specific scores were adapted for HFrEF medications and include two 5-item subscale scores ranging from 5 to 25. Higher BMQ-Specific subscale scores indicate stronger beliefs that their HFrEF medications are necessary (*Specific-necessity*) or greater concerns that their HFrEF medications lead to long-term toxicity and adverse effects (*Specific-concern*).^17^

Key informant in-depth interviews were conducted in-person, by phone, or via videoconference. We used semistructured interview guides based on the Consolidated Framework for Implementation Research (CFIR) v2.0, an implementation science framework that employs a systematic approach to understand the factors influencing innovation implementation.^18^ Purposeful and convenience sampling were used to recruit: 1) patients with HFrEF from diverse racial backgrounds during outpatient visits or hospital admissions; and 2) providers with different health care roles at each study site. Interviews were audio-recorded, transcribed, and analyzed using Dedoose (v9.0.107, SocioCultural Research Consultants LLC). We employed a two-phase approach to analysis: first deductive, then inductive content analysis using CFIR v2.0.^19^ The codebook, themes, and relationships were derived using a constant comparative method. Interviews continued until thematic saturation was achieved, defined as the absence of new codes for 3 consecutive interviews and consensus by coders (J.C.C. and C.D.).^20^^,^^21^ To ensure trustworthiness, we triangulated data from a diverse group of patients and health care providers and applied intentional self-reflexivity following Milner’s approach.^22^

After identifying key determinants affecting implementation of the HFrEF polypill, we used the updated CFIR-Expert Recommendations for Implementing Change (CFIR-ERIC) Matching Tool to select relevant strategies for each contextual barrier.^23^^,^^24^ This tool provides a structured approach to select from 73 implementation strategies aligned with specific CFIR constructs. Strategies are classified as “Level 1” or “Level 2” endorsement if ≥50% or 20% to 49.9% of 169 surveyed experts in implementation science, respectively, endorsed the strategy for a specific CFIR construct.^23^ We then used themes on strategies to support HFrEF care or the HFrEF polypill to further refine which strategies were included in the HFrEF polypill implementation bundle. Finally, we used mechanism mapping to develop a directed acyclic graph, hypothesizing how this implementation bundle could improve HFrEF care through multiple interacting pathways and mediators.^25^, ^26^, ^27^ The relationships between constructs were informed by both literature review and themes on strategies to support HFrEF care and implementation of the HFrEF polypill.

## Results

### Key informant in-depth interviews

#### Participant characteristics

We initially contacted 57 patients and health care providers, and subsequently interviewed 31 participants (Table 1). Key informant in-depth interviews with 9 patients and 22 health care providers (including primary care providers, hospitalists, cardiologists, and nurse practitioners) ranged from 20 to 75 minutes, with most lasting between 40 and 50 minutes. BMQ subscale scores indicated that patients on average disagreed that medications are harmful, were uncertain whether doctors overprescribe medications, agreed the adverse effects of HFrEF medications were concerning, and agreed their HFrEF medications were necessary (Table 1).

**Table 1:** Participant Demographic Characteristics of Patients and Providers Who Completed Key Informant In-Depth Interviews (N = 31)

Patients (n = 9)	
Age, y (mean, SD)	57.6 (16.5)
Sex	
Female	4 (44.4)
Male	5 (55.6)
Race^a^	
Asian	1 (11.1)
Black or African American	3 (33.3)
White	3 (33.3)
Did not report	2 (22.2)
Ethnicity	
Hispanic or Spanish or Latin origin	1 (11.1)
Not Hispanic or Spanish or Latin origin	8 (88.9)
Highest level of education	
Secondary/high school	3 (33.3)
Some college/university	2 (22.2)
Associate/technical degree	1 (11.1)
Postgraduate degree	3 (33.3)
Employment status	
Employed full time	2 (22.2)
Retired	2 (22.2)
Disabled	3 (33.3)
Supplemental Security Income	1 (11.1)
Not employed	1 (11.1)
Health insurance^a^	
Individually purchased private	1 (11.1)
Medicare	5 (55.6)
Medicaid	6 (66.7)
Other	1 (11.1)
Modified belief about medicines questionnaire^b^	mean (SD)
Adapted General-Harm	9.0 (2.14)
Adapted General-Overuse	11.0 (1.77)
Adapted Specific-Concern	18.6 (3.61)
Adapted Specific-Necessity	20.2 (3.46)
Health Care Providers^c^ (n = 22)	
Age, y (mean, SD)	46.1 (11.0)
Clinical experience, y (mean, SD)	12.2 (9.0)
Sex	
Female	13 (59.1)
Male	8 (36.3)
Nonbinary	0 (0)
Prefer not to say	1 (5.3)
Race^a^	
American Indian or Alaska Native	0 (0)
Asian	8 (42.1)
Black or African American	2 (10.5)
Native Hawaiian or other Pacific Islander	0 (0)
White	9 (47.4)
Other	0 (0)
Ethnicity	
Hispanic or Spanish or Latin origin	1 (5.3)
Not Hispanic or Spanish or Latin origin	18 (94.7)
Primary health care role	
Primary care provider	6 (27.3)
Hospitalist	4 (21.1)
General cardiologist	7 (31.8)
Advanced heart failure cardiologist	3 (13.6)
Cardiology advanced practice provider	2 (9.1)
Health care communities^a^	
Urban	19 (100.0)
Suburban	6 (31.6)
Rural	3 (15.8)
Health care setting^a^	
Private practice	1 (5.3)
Academic medical center	18 (94.7)
Long-term care facility	1 (5.3)
Government affiliated health care system	1 (5.3)
Federally qualified health center	1 (5.3)

Values are n (%).

a Respondents were allowed to select multiple answers.

b 2 patients did not complete one item from the Belief about Medicines Questionnaire and their responses for that subscale was excluded from analysis.

c Of the 22 health care providers who participated in interviews, 3 did not complete the demographic survey, and are only represented in the “Healthcare role” and “Sex” sections. The other sections (age, years in practice, race, ethnicity, health care communities, and health care setting) include the 19 participants for which these data are available.

**Figure 1:**
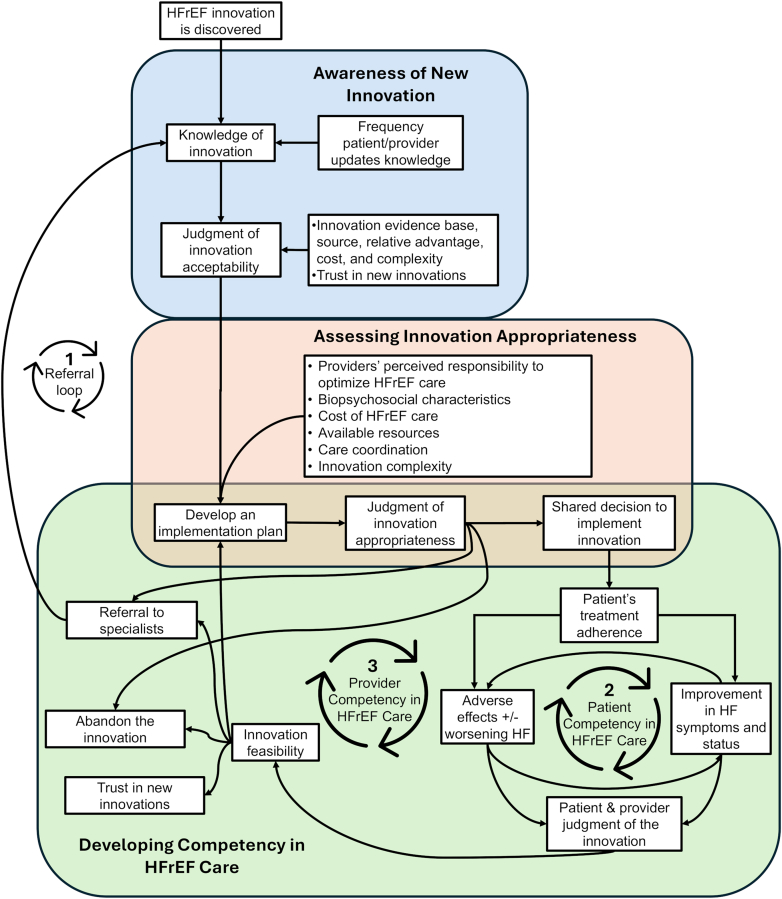
**Conceptual Model Describing How Stakeholders Implement New HFrEF Innovations** This conceptual model describes how HFrEF innovations are adopted in 3 stages based on thematic analysis of key informant in-depth interviews: 1) obtaining awareness of the innovation; 2) assessing the innovation’s appropriateness; and 3) developing competency in HFrEF care and implementing the innovation while managing patients with HFrEF. Arrows indicate direction of influence and looping arrows describe reinforcing cycles. HFrEF = heart failure with reduced ejection fraction.

Four themes emerged from the study describing factors that would influence implementation of HFrEF polypills. We identified the *current state of HFrEF care* as an organizing theme, illustrating how existing barriers to HFrEF care influence implementation of HFrEF polypills. Three additional themes describe the processes and factors that may influence HFrEF innovation implementation: 1) *awareness of new innovations* (how patients and providers become aware of HFrEF innovations); 2) *assessing innovation appropriateness* (contextual factors that affect whether an innovation is trialed and adopted); and 3) *building competency in HFrEF care* (how patients and providers develop the knowledge, skills, and attitudes necessary to manage HFrEF and HFrEF innovations). Based on our thematic analysis, Figure 1 illustrates a conceptual model depicting how patients and providers adopt and implement new innovations such as HFrEF polypills (Supplemental Results 1, Supplemental Figure 1). We present exemplary quotes describing how determinants influence both HFrEF care and implementation of HFrEF polypills in Table 2. Quotes in Table 2 have been modified for ease of reading without changing content or meaning. There were no significant differences in themes generated from different prescriber types (eg, cardiologists vs noncardiologists) or study sites (St. Louis vs San Francisco). Because patients had limited understanding of: 1) how acceptable and appropriate an innovation is; 2) available resources to support implementation; and 3) how a HFrEF polypill would influence their treatment, these themes were largely formed from prescriber perspectives. For these areas, patients would defer to their health care team’s recommendations and trust them to act in their best interest.

**Table 2:** Exemplar Quotes Illustrating Parallel Barriers Affecting HFrEF Care and Implementation of the HFrEF Polypill

Key Determinants	Exemplar Quotes for HFrEF Care	Exemplar Quotes for HFrEF Polypill Implementation
Theme 1: Awareness of new innovations		
1. Trust in new innovations	[Providers] can sense bullshit and spin from pretty far away. And so, [a new therapy] isn’t successful or isn’t actually worth it, but it's forced down from a top-down approach. […] I mean even a lot of cardiologists right now think about the [2 academic cardiology associations] as basically being in the pockets of pharma. (General Cardiology 1)	I've had different doctors in the hospital. I live in a small town and they always want to try something different, and I finally always just tell them, "You call Dr [heart failure specialist] and ask if it's okay, and then I'll take it [the HFrEF polypill]." (Patient 7)
2. Delayed adoption of best practices in HFrEF care	There is a reluctance to start [HFrEF GDMT] that hasn't been started in the outpatient setting. Sometimes I'll see in the notes “defer to primary or primary cardiologist” … Or they came off the cards service and [HFrEF GDMT] weren't started and I don’t know why… Especially when they're plugged into subspecialty care who should in theory be advancing the guideline driven therapy, who am I as an internist to deviate from what the last cardiologist said to do? (Hospitalist 3)	I would not include the SGLT2i and Entresto [in the HFrEF polypill] just because I personally don't have that much experience in titrating those[…] Some patients may not meet criteria for those 2 drug classes, whereas they might meet criteria for the other ones. […] So maybe just giving us as a community time to get accustomed to that and incorporate that into our practice. […] I have seen people really defer to other specialists or endocrinologists instead of taking initiative as a cardiologist to start someone on an SGLT2i. (General Cardiology 7)
Theme 2: Assessing innovation appropriateness		
3. Patient biopsychosocial characteristics influence provider practices	And I thought, hmm, I wonder why I'm not doing that [prescribing an ARNI]. And I realized that what I was doing when I had a patient in front of me, I would use what at that time I called my clinical judgment. And I would decide, they probably wouldn't tolerate the medicine. Their blood pressure will get too low. They're old, they probably won't tolerate it, blah, blah, blah. (Heart Failure 4)	Another thing is that local people will be hesitant to prescribe [the HFrEF polypill] to someone that's from a lower socioeconomic background that has unstable follow up because they might not be able to check their labs, update how they're feeling, see if they are getting too hypotensive, or something else. They might say, “Hey, there's too much downside risk, I'm not going to prescribe it in this patient.” And so, I think a potential big component is whether people will be too scared to prescribe it because of uncertain follow up. (General Cardiology 1)
4. Time constraints	I think the biggest issue with counseling is that our patients have many complex medical issues. So time, I think, is the biggest barrier, because patients come in, you're following them up for their HFrEF, but then you're also having to address their diabetes, their osteoarthritis, their hyperlipidemia. And so sometimes the counseling part turns into a one-liner, “I'm so glad you're taking these,” or, “you're not taking them. I think it's a great idea for you to take them because it's good for your heart.” And then that's it, which is better than nothing. If you're really trying to make the patient recognize how important those medications are, that one sentence probably isn't going to be enough. (PCP 3)	Things that would make me shy away from [the HFrEF polypill] is just tracking out side effects. So if I knew that they might get in trouble because there's this nuance to this… it comes down to how much time do I want to spend building a mitigation plan to take care of that potential downside. So if that plan takes too long for me to build or requires me to do some customization with primary [care physicians], that's not possible. (Hospitalist 1)
5. Insufficient support from nonphysician health care providers	In my clinic, I'm not going to have people coming back every 2 weeks for medication up titration because that's an inefficient use of my time. I don't have sufficient mid-level provider support. I do think the most effective way to do that is to put pharmacists and mid-level providers in charge of [up titrating medications], because they follow the guidelines, they do what the guidelines say, and they get patients to good doses. (Heart Failure 4)	So, if you're going to be trying to get [the HFrEF polypill] out into some of the places where I work, [primary care setting for underserved patients], I don't have a physician's assistant. I've got a medical assistant who graduated from high school of varying quality. And the highest, most knowledgeable person in the clinic, other than the physician, is most likely to be an LPN or an RN of varying quality. (PCP 1)
6. Limited access to cardiovascular specialists	One of our issues in the clinic is we really want them to see a cardiologist, but there are people who don't actually have the cardiology follow up that we think they should have. (PCP 2)	Make sure that the physicians who are treating those patients, especially the primary care physicians, know about [the HFrEF polypill] and have access to it. Because not all patients have access to cardiologists, but hopefully they have access to primary care physicians since they're the first line of care. (Heart Failure 5)
7. System tools are not optimally implemented to support HFrEF care	There's so much that gets lost in Epic. It's so hard to search for adverse effects. If there were a standard place to look or a standard template to understand the adverse events or the intolerances because sometimes [a HFrEF GDMT] doesn't make it into the allergy list or it's not a true allergy. (Hospitalist 3)	One might start with the polypill at [one institution] and they go to [another institution] and we can't fully see the medication reconciliation or it's not updated. That doctor then doesn't realize that polypills have been started and they continue to titrate up things individually. Then the patient ends up getting a little bit too much medication in their system. (General Cardiology 1)
8. Cost of HFrEF care	I think that doctors don't have that concern about whether their patients can pay for their medicine. They'll just write out the prescription and say, well, then it's up to you. (Patient 6)	The whole philosophy of the polypill is to make it broadly accessible and really, really convenient and cheap. It can't be like an expensive convenience thing […] I think that if we end up putting in a bunch of expensive stuff in there, it's going to really limit the access. (General Cardiology 8)
9. Institutional constraints	Some pharmacies didn’t carry the medicines that the doctor had prescribed for me so I had to search around and ask different pharmacies. And then certain pharmacies that did have it told me how much it costed and they said my Medicaid wouldn’t cover it. So I was like, how am I supposed to get the medicine? (Patient 1)	You usually don't have access to the newest stuff, because your population is going to be Medicaid or self-pay, or whatever the equivalent is. So, if the mini pill comes out and it's a hot new item, I probably am not going to have any access to it for a while. Pharmacists may not have heard about it; the clinic pharmacy may not carry it. My patients may not be able to get it. So, we may lag for a while. (PCP 1)
Theme 3: Building competency in HFrEF care		
10. Inconsistent treatment adherence	It was hard in the sense of just knowing that – some of the medicines I had to take twice a day, I just didn’t want to believe that I should be taking that much medicine. It was all in my brain, you know, that I didn’t – saying, I’m not sick. I just kept saying, I’m not claiming it; I’m not claiming none of that because I’m not sick. But I was sick; I was sick… eventually, I had got to the point where I take the medicine then I don’t. (Patient 2)	For some patients, they'll take their morning pills, maybe not take this pill or that pill, but then they're getting at least some of them. If they run out of their medications and don't go to the pharmacy to pick them up, […] they may be out of their carvedilol for a week, but at least they're still on their lisinopril and their spironolactone. But if it's a polypill and they run out and don't pick it up for a week, then they're going to miss all of their meds for a week. (PCP 2)
11. Poor care coordination	If your refills are not coordinated in time, maybe one is going to be out in the middle of the month and the other is going to be out not until the end of the month, they may get some but not the others. […] They may not realize that they're consistently forgetting about one. I've had patients come and when we review their medications, they'll say, I haven't had that one for X amount of time. And I'll say, did your doctor stop it? And they’re like, no. They just stopped giving it to me. And I think what happens is, if you stop asking for refills, then the pharmacy isn't going to wake up and call you and say, you need a refill, right? So, the patient kind of loses track of their medications. (PCP 1)	We don't have really any mechanism for deprescribing. So if we're taking over as a polypill prescriber, I think there would be risk for med errors. If they're still getting calls from [a pharmacy] like “hey, your refill for your ARNI is available,” they're leaving with this polypill in hand and still getting messages to take the individual medicines. (PCP 4)
12. Therapeutic inertia	It's not uncommon when we start a SGLT2i, one may see a little creatinine elevation with the ARNI or ACEi or ARB. Or we see a little elevated potassium when somebody adds an aldosterone receptor blocker that’s tolerable. This is where we see plenty of patients whose practitioners stop some medications very early because they perceived a bump in creatinine or mild bump in potassium when it's not necessary. (General Cardiology 6)	I feel like if you are on polypill, there might be more hesitancy to titrate up. Like if you come in and your blood pressure is 110 and the physician thinks once they titrate up they may be titrating on multiple medications, they may hesitate to titrate up rather than if they were just titrating one. (General Cardiology 2)

ACEi = angiotensin-converting enzyme inhibitor; ARB = angiotensin receptor blocker; ARNI = angiotensin receptor/neprilysin inhibitor; GDMT = guideline-directed medical therapy; HFrEF = heart failure with reduced ejection fraction; LPN = licensed practical nurse; RN = registered nurse; SGLT2i = sodium-glucose cotransporter-2 inhibitors; other abbreviation as in Table 1.

#### Organizing theme: current state of HFrEF care

Although participants viewed HFrEF polypills as a practical strategy to improve medication adherence and increase the use of HFrEF GDMT, they expressed uncertainty about whether HFrEF polypills could be implemented safely and provide hypothesized benefits. For example, health care providers who are hesitant to prescribe a sodium-glucose cotransporter-2 inhibitor were also reluctant to prescribe an HFrEF polypill containing a sodium-glucose cotransporter-2 inhibitor. Therefore, current barriers that impair the adoption of HFrEF innovations may also influence the adoption of an HFrEF polypill. Many participants believed the availability of an HFrEF polypill alone would not significantly alter the current state of HFrEF care and emphasized the need for supporting implementation strategies to address existing barriers in HFrEF care.

#### Theme 1: awareness of new innovations

Convenience and availability of trusted sources affected how frequently patients and providers updated their knowledge of HFrEF innovations. Many providers stated that they discover innovations like an HFrEF polypill through scheduled educational conferences and observing the practice patterns of trusted specialists. Similarly, most patients became aware of an HFrEF innovation once recommended by their health care provider and would only trial an HFrEF polypill if they trusted the health care provider (Determinant 1, Table 2). Some cardiologists actively updated their knowledge of innovations in HFrEF care by continuously reviewing the literature and seeking additional educational opportunities. After gaining awareness of innovations, stakeholders make a judgment of the innovation’s acceptability based on information provided. Although this passive approach to discovering HFrEF innovations leads to delayed awareness, prescribers emphasized the importance of disseminating through trusted local representatives who appreciate the prescriber’s and patient’s unique context (Determinant 2, Table 2).

#### Theme 2: Assessing innovation appropriateness

Although the HFrEF polypill may appear acceptable under theorized conditions, both patients and prescribers were concerned about how an HFrEF polypill would be used under real-life conditions. While the HFrEF polypill-based strategy may simplify GDMT initiation and promote medication adherence, combination therapies may also complicate HFrEF GDMT titration. Therefore, participants would assess the feasibility of a personalized implementation plan—one that considers each patient’s biopsychosocial characteristics and available resources—when determining who should be prescribed an HFrEF polypill. Prescribers were more hesitant to initiate and uptitrate an HFrEF polypill in patients with more severe biopsychosocial factors, a group already less likely to receive timely and optimal HFrEF GDMT (Determinant 3, Table 2). Strategies that increase resources like time, supporting staff, access to specialists, supporting health care tools/processes, health care affordability, and other institutional resources would promote uptake and sustained use of the HFrEF polypill (Determinant 4-9, Table 2). If participants perceived the implementation plan to be unsafe or impractical, participants would likely abandon the innovation, defer GDMT initiation to future visits, or refer to a specialist (Cycle 1 in Figure 1).

#### Theme 3: Building competency in HFrEF care

Many patients struggled to manage their HFrEF after their initial diagnosis because they did not believe their diagnosis or lacked sufficient knowledge to manage their condition. As a result, adhering to recommendations and coordinating with providers were challenging (Determinant 10 and 11, Table 2). Patients then experienced cycles of heart failure exacerbations and recovery as they struggled to adopt healthy behaviors. As patients realized the positive impact of medication adherence on their symptoms, quality of life, and HFrEF status, they took ownership of managing their health (Cycle 2 in Figure 1). Patients with adverse socioeconomic conditions, poor health literacy, and less motivation often developed more severe HFrEF symptoms and experienced more HFrEF exacerbations before they were able to manage their illness.

Similarly, providers gained competency in providing guideline-directed HFrEF care by connecting how their prescribed HFrEF treatment plans affected their patient’s symptoms and HFrEF status (Cycle 3, Figure 1). After trialing an innovation, patients and providers must integrate multiple factors, such as medical comorbidities and treatment adherence, to evaluate whether innovations like the HFrEF polypill provided greater benefit than side effects. Patients and providers who are more cautious about minor changes would be more hesitant to adopt and quicker to discontinue the HFrEF polypill (Determinant 12, Table 2). If an innovation seemed impractical for HFrEF care, providers chose to either continue trialing the innovation for other patients, abandon the HFrEF innovation altogether, or refer the patient to a specialist (Cycle 1, Figure 1).

### CFIR-ERIC mapping tool

Using the CFIR-ERIC Matching Tool, we identified 13 Level 1 endorsed strategies and 51 Level 2 endorsed strategies to address 15 CFIR-based barriers. We further refined our selection of strategies using themes from key informant in-depth interviews describing strategies to support implementation of HFrEF polypills (Table 3, Supplemental Table 1, Supplemental Results 1). We organized these implementation strategies into 7 domains: facilitation, staged implementation plan, adapting and tailoring the implementation program to stakeholders, quality management, knowledge broker, team-based care, and the HFrEF polypill (Table 4).

**Table 3:** Identified Strategies Using the CFIR-ERIC Matching Tool for a Multilevel HFrEF Polypill Implementation Bundle to Improve HFrEF Care

Adapted Determinants for a HFrEF Polypill^a^	CFIR Construct	Level 1 Strategy	Selected Level 2 Strategy
Awareness of new innovations			

1. Trust in new innovations	Culture	• Identify and prepare champions	• Conduct local needs assessment • Facilitation • Conduct local consensus discussions • Identify early adopters • Recruit, designate, and train for leadership
2. Delayed adoption of best practices in HFrEF care	Access to knowledge and information	• Conduct educational meetings • Develop educational materials • Distribute educational materials	• Provide local technical assistance • Use advisory boards and workgroups • Facilitate relay of clinical data to providers • Involve patients/consumers and family members • Use mass media • Shadow other experts • Conduct ongoing training
	Engaging: deliverers	• Identify and prepare champions	

Assessing innovation appropriateness			

3. Patient biopsychosocial characteristics influence provider practices	Tension for change	• None	• Assess for readiness and identify barriers and facilitators • Identify and prepare champions • Facilitate relay of clinical data to providers • Involve patients/consumers and family members
4. Time constraints 5. Insufficient support from nonphysician health care providers 6. Limited access to cardiovascular specialists	Available resources	• Access new funding	• Capture and share local knowledge • Change physical structure and equipment • Fund and contract for clinical innovation • Develop resource sharing agreements
7. System tools are not optimally implemented to support HFrEF care			
8. Cost of HFrEF care			
9. Institutional constraints			

Building competency in HFrEF care			

10. Inconsistent treatment adherence	Engaging: recipients	• Prepare patients/consumers to be active participants • Involve patients/consumers and family members • Intervene with patients/consumers to enhance uptake and adherence	• Alter patient/consumer fees • Capture and share local knowledge • Conduct educational outreach visits • Create a learning collaborative • Use advisory boards and workgroups • Visit other sites
11. Poor care coordination	Partnerships and connections	• Build a coalition • Develop academic partnerships • Promote network weaving	
12. Therapeutic inertia	Executing	• None	• Develop a formal implementation blueprint • Develop and implement tools for quality monitoring • Facilitation • Provide local technical assistance • Use an implementation adviser
The HFrEF polypill^b^			

13. Adaptability of HFrEF polypill to patients’ unique needs	Innovation adaptability	• Promote adaptability	• Assess for readiness and identify barriers and facilitators • Capture and share local knowledge • Conduct cyclical small tests of change • Conduct educational meetings • Develop a formal implementation blueprint • Identify and prepare champions • Provide ongoing consultation • Stage implementation scale up • Increase demand • Organize clinician implementation team meetings
14. Complexity to safely titrate HFrEF polypill	Innovation complexity Innovation relative advantage	• None • None	
15. HFrEF polypill cost	Innovation cost	• Access new funding	• Place innovation on fee-for-service lists/formularies • Alter incentive/allowance structures • Alter patient/consumer fees • Use other payment schemes • Make billing easier

CFIR = Consolidated Framework for Implementation Research; ERIC = Expert recommendations for implementing change; other abbreviation as in Table 2.

a Barriers for a HFrEF polypill implementation strategy are adapted from barriers within Table 2.

b Barriers specific to the HFrEF polypill are previously reported.^7^

**Table 4:** Potential Core Components of a Multilevel HFrEF Polypill Implementation Bundle

Strategy	Components
1. Facilitation	• Implementation strategy mapping to describe program objectives, determinants, outcomes, and theory-informed strategies • Engage relevant stakeholders • Identify and cultivate champions and early adopters • Disseminate practice insights from other providers and facilities • Just-in-time assistance for individual patients and providers
2. Staged implementation plan	• Scale implementation program stratified by health care provider and clinical settings
3. Adapting and tailoring implementation program to stakeholders and contextual factors	• Assess for readiness and identify barriers and facilitators • Focus groups with patients and providers to design, adapt, and improve care delivery and polypill implementation • Promote network weaving • Change physical structure and equipment • Continuously re-evaluate implementation program
4. Quality management	• Develop tools to continuously monitor process and outcome measures • HFrEF GDMT registry/dashboard • Timely and strategic GDMT EMR alerts for initiation, medication interactions, and discontinuation
5. Knowledge broker	• Develop and distribute HFrEF polypill educational materials and curricula to patients and providers
6. Team-based care, engagement, and feedback	• Develop a coalition of providers and patients to provide feedback and insight on the intervention to the implementation team, providers, and patients
7. HFrEF polypill	• Promote fixed-dose combinations in clinical protocols and guidelines • Increase number of HFrEF polypill formulations • Promote broad insurance coverage • Promote inpatient and outpatient pharmacy access to HFrEF polypill • Provide cost assistance programs

EMR = electronic medical record; GDMT = guideline-directed medical therapy; other abbreviation as in Table 2.

### Mechanism mapping

**Figure 2:**
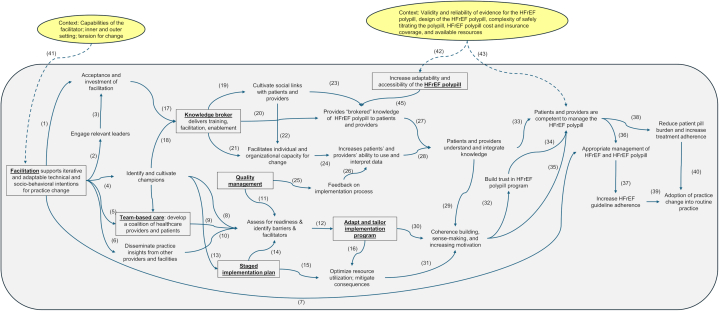
**Directed Acyclic Graph of a Multilevel HFrEF Polypill Implementation Bundle** We illustrate how a multilevel HFrEF polypill implementation bundle centered around facilitation could improve patient outcomes with a directed acyclic graph based on literature review and themes on strategies to support HFrEF care and implementation of the HFrEF polypill. Arrows indicate direction of influence. The 7 potential core components of the multilevel bundled intervention are represented as black boxes. Contextual effects are represented as yellow ellipses pointing to specific nodes in the chain of events. Abbreviation as in Figure 1.

After selecting *which* strategies should be included in the multilevel HFrEF polypill implementation bundle for HFrEF, we used mechanism mapping to develop a directed acyclic graph that hypothesizes *how* the implementation bundle could improve HFrEF care through multiple interacting pathways and mediators. We used themes describing strategies to support HFrEF care and HFrEF polypill implementation, and existing literature to design a directed acyclic graph (Figure 2, Supplemental Results 1).^26^, ^27^, ^28^, ^29^, ^30^, ^31^, ^32^ In this conceptual model, facilitation is the primary strategy for the multilevel HFrEF polypill implementation bundle. Facilitators would engage with stakeholders to provide technical expertise and guide HFrEF polypill implementation through 6 pathways: 1) promoting organizational acceptance and further investment in facilitation (Arrow #1, Figure 2); 2) engaging relevant leaders and stakeholders (Arrow #2&3, Figure 2); 3) cultivating champions (Arrow #4, Figure 2); 4) developing a diverse coalition of providers and patients to support implementation (Arrow #5, Figure 2); 5) disseminating practice insights from other sources (Arrow #6, Figure 2); and 6) providing technical assistance (Arrow #7, Figure 2). The facilitator then incorporates stakeholder feedback and quality management data to assess readiness for change and identify barriers and facilitators for adoption (Arrow #8-11, Figure 2). A staged implementation approach allows adaptive and iterative improvements based on identified barriers to optimize resource utilization and mitigate consequences (Arrow #12-16, Figure 2).

Facilitators would also act as knowledge brokers, teaching patients and providers how to manage the HFrEF polypill, foster rapport, and cultivate individual skills (Arrow #17-23, Figure 2). Quality management and educational initiatives will enhance patient and provider capacity (Arrow #24-26, Figure 2), foster a shared understanding, cultivate trust and motivation to maintain implementation, and empower effective participation in the HFrEF polypill implementation process (Arrow #27-35, Figure 2). Improved competence in HFrEF care would then promote provider HFrEF guideline adherence and patient treatment adherence (Arrow #36-38, Figure 2), fostering adoption and routine use of HFrEF polypills (Arrow #39-40, Figure 2).

Various contextual factors could influence the impact of an HFrEF polypill-based strategy. A facilitator’s ability to engage stakeholders and lead change will depend on their skills, factors within the inner and outer setting, and tolerability of the current HFrEF care paradigm (Arrow #41, Figure 2). The HFrEF polypill’s design, adaptability, and cost will influence the feasibility, penetrance, and sustainability of a polypill-based approach to HFrEF (Arrow #42&43, Figure 2).

## Discussion

Our findings describe the perspectives of patients and health care providers on determinants that may influence the implementation of HFrEF polypills. The organizing theme that emerged from the analysis was that the current state of HFrEF care, including barriers, must be addressed for successful implementation of a polypill-based strategy for HFrEF. We provide a conceptual model that illustrates how patients and providers: 1) become aware of new HFrEF innovations; 2) decide whether an HFrEF innovation is appropriate; and 3) build competency in managing patients with HFrEF and implementing HFrEF innovations. We then developed and described how a multilevel HFrEF polypill implementation bundle tailored to identified determinants could improve HFrEF care (Central Illustration).

**Central Illustration:**
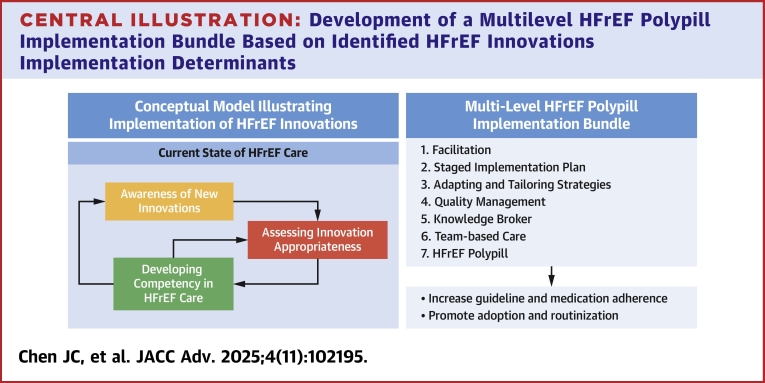
**Development of a Multilevel HFrEF Polypill Implementation Bundle Based on Identified HFrEF Innovations Implementation Determinants** We describe an HFrEF implementation bundle to address identified determinants that influence implementation of HFrEF innovations that emerged as four themes from key informant in-depth interviews with 31 patients and health care providers at 2 academic health care systems. Strategies were selected using the Consolidated Framework for Implementation Research-Expert Recommendations for Implementing Change Matching Tool and themes from in-depth interviews. Abbreviation as in Figure 1.

Our conceptual model for HFrEF innovation implementation closely aligns with the innovation-decisions model and offers additional insights into the consequences of delayed adoption of HFrEF innovations.^33^ While future randomized controlled trials may demonstrate that the HFrEF polypill benefits patients *on average*, both patients and providers still face the uncertainty of whether the HFrEF polypill will be effective for a given *individual*. Patients with HFrEF have high 5-year mortality rates and often experience heart failure exacerbations despite their best efforts to adopt challenging lifestyle modifications and their provider’s attempts to follow evidence-based HFrEF care.^1^ As a result, patients and providers must consider multiple factors (eg, the patient’s biopsychosocial characteristics and feasibility of a polypill-based approach) when evaluating whether to adopt or reject the HFrEF polypill. Delays in adopting such innovations often lead to more frequent HFrEF exacerbations, the development of additional comorbidities, and increased intolerance to innovations like HFrEF GDMT. To address these challenges, hybrid-effectiveness randomized controlled trials that evaluate both the implementation process along with effectiveness and safety of HFrEF polypills from the outset will be critical for accelerating equitable integration of polypill-based strategies into HFrEF care.^34^

Our findings are consistent with prior studies applying implementation science to increase HFrEF GDMT-prescribing practices. We highlight how determinants across multiple domains—including individual, innovation, inner and outer settings, and the implementation process—interact to hinder the optimal use of HFrEF GDMT and other evidence-based innnovations.^18^^,^^35^ This complex network of determinants likely contributes to the limited effectiveness, incomplete adoption, and variable success of interventions that only target a single domain or too few domains.^11^^,^^36^ To achieve widespread equitable implementation and sustained improvements in patient outcomes, interventions must be strategically developed, adapted to contextual factors, and described in detail to promote dissemination. Implementation studies describing the impact of a patient activation tool in the EPIC-HF (Electronically Delivered, Patient-Activation Tool for Intensification of Medications for Chronic Heart Failure with Reduced Ejection Fraction) trial and a clinician decision support tool for HFrEF demonstrate how implementation science can be used to rigorously investigate how determinants contribute to the treatment gap, strategically develop and implement strategies, and evaluate their impact on service, implementation, and clinical outcomes.^37^^,^^38^ Even implementation studies that do not meet their primary outcome, like the DASH-HF (Dashboard Activated Services and Telehealth for Heart Failure) pilot pragmatic randomized implementation trial, provide valuable insights by reporting implementation outcomes that can guide the refinement of interventions to better address modifiable contextual factors.^39^^,^^40^ Leveraging implementation science methodologies to improve HFrEF care, like designing for dissemination and implementation mapping, will provide more effective, practical, sustainable, and scalable interventions that can be adapted to new populations, geographic regions, and unique contexts.^25^^,^^41^

Recognizing that implementation is dependent on context, the multilevel HFrEF polypill implementation bundle provides multiple evidence-based strategies that can be further tailored to the context for HFrEF polypill implementation. For example, facilitation strategies may be relevant to HFrEF polypill trials in both the United States (NCT04633005, NCT06029712) and Sri Lanka (SLCTR/2024/003, NCT06831864); whereas quality management strategies incorporating electronic medical record alerts or dashboards are less relevant in health systems without robust electronic medical records.^12^, ^13^, ^15^ Although implementing a multilevel bundled intervention to support an HFrEF polypill intervention may not be feasible in all settings, this analysis provides a suite of strategies that investigators can use to create and further tailor their own HFrEF polypill implementation bundle to the contexts they operate in.

Further research is necessary to understand how HFrEF polypills should be designed and supported with implementation strategies to maximize impact on patient outcomes. An ongoing multicenter discrete choice experiment will provide additional insights on which HFrEF polypill design characteristics are most important to cardiologists.^42^ Ongoing trials testing different HFrEF polypill combinations will generate evidence necessary to understand the safety, effectiveness, and implementation process of HFrEF polypills.^12^, ^13^, ^14^, ^15^ Further studies are needed to gather perspectives of nonacademic health care providers, patients from underserved populations, the pharmaceutical industry, and payers. These collective efforts will ensure the safe, effective, and equitable implementation of HFrEF polypills.

### Study Limitations

This study has several limitations. While CFIR v2.0 offers numerous constructs to guide needs assessments, it is often used superficially to identify determinants without fully exploring how interactions among those determinants contribute to the problem. Our key informant in-depth interviews sought to explore these interconnections, and we have presented these insights in our conceptual model to build on previously published analyses and Implementation Research Logic Model for a polypill-based strategy for HFrEF.^16^ The CFIR-ERIC Matching Tool also has key limitations.^23^^,^^43^^,^^44^ This tool categorizes complex barriers as nonspecific CFIR constructs before suggesting endorsed strategies. The barriers consequently lose precision and context as CFIR constructs, resulting in many implementation strategies offered that may not be relevant for the specific barrier of interest. To overcome this limitation, we incorporated stakeholder-recommended strategies to refine which strategies are included in the multilevel HFrEF polypill implementation bundle and how they should be implemented. Ultimately, CFIR v2.0 and the CFIR-ERIC Matching Tool serve as guides; their effectiveness depends on how comprehensively and rigorously they are applied to address identified determinants.^18^^,^^23^ Our findings have limited generalizability because our participants were primarily health care providers from academic centers and patients who perceived their HFrEF medications as necessary for maintaining their health and were younger than the average age of patients with HFrEF.^45^ A key strength is the rigor of our approach, which combined multiple implementation science frameworks and triangulated insights from a diverse group of stakeholders.

## Conclusions

We use a systematic approach grounded in implementation science to develop a multilevel HFrEF polypill implementation bundle, with the goal of improving HFrEF care in undertreated populations globally. This conceptual model will inform future randomized controlled trials to evaluate the effectiveness of a polypill-based strategy to improve HFrEF care.

Perspectives **COMPETENCY IN SYSTEMS-BASED PRACTICE:** We identified 12 barriers that impact current HFrEF care and provide a conceptual model illustrating how those barriers affect uptake, implementation, and continued use of new HFrEF innovations.

**TRANSLATIONAL OUTLOOK 1:** Participants believed an HFrEF polypill alone would not significantly change HFrEF care because the current barriers to optimal HFrEF care will impede implementation of a polypill-based strategy for HFrEF.

**TRANSLATIONAL OUTLOOK 2:** Participants described multiple implementation strategies that could promote safe, equitable, and effective implementation of a polypill-based strategy for HFrEF.

## Funding support and author disclosures

This work was funded in part by a UCSF CAPS-HIV Innovative Grant (P30MH062249; support for Drs DeJong, Durstenfeld, and Hsue) and a UCSF CFAR/ARI HIV Boost Award in San Francisco, California (support for Drs DeJong, Durstenfeld, and Hsue). Dr Agarwal is funded by NIH/NHLBI Grant R00HL157687 (Bethesda, MD) and has received funding from Grant 2020144 from the 10.13039/100000862Doris Duke Charitable Foundation (New York, NY). Dr Durstenfeld is funded by 10.13039/100000002NIH/10.13039/100000050NHLBI grant K23HL172699 (Bethesda, MD). Dr DeJong was funded by an ACC/ABC Merck Research Fellowship Award (Washington, DC). Dr Hsue is funded by 10.13039/100000002NIH/10.13039/100000060NIAID Grant K24AI112393 (Bethesda, MD). Dr Huffman has received travel support from the American Heart Association and World Heart Federation; has an appointment at The George Institute for Global Health, which has a patent, license, and has received investment funding with the intent to commercialize fixed-dose combination therapy through its social enterprise business, George Medicines. Drs Huffman and Agarwal plan to submit patents for heart failure with reduced ejection fraction polypills. Dr DeJong’s spouse is employed by and holds stock in iRhythm Technologies. Dr Durstenfeld has received consulting fees from Merck unrelated to this manuscript. Dr Hsue has received honoraria from Gilead, Pfizer, Merck, Genentech, and grant support from Excision Biotherapeutics, Scientific Advisory Board, Marea Therapeutics, all unrelated to the manuscript. The content is solely the responsibility of the authors and does not necessarily represent the official view of the NIH. All other authors have reported that they have no relationships relevant to the contents of this paper to disclose.
