# Supplementary material for: Developing a Multilevel Polypill Implementation Bundle for Patients With Heart Failure With Reduced Ejection Fraction
Source: JACC Adv. 2025 Oct 8;4(11):102195. doi: 10.1016/j.jacadv.2025.102195 (PMC12717559; doi:10.1016/j.jacadv.2025.102195)
Supplement: Supplemental Material [file mmc1.docx]

**Supplemental Appendix**

[Supplemental Methods. Beliefs about medicines questionnaire 2](#_Toc202822257)

[Supplemental Results. Thematic analysis 0](#_Toc202822258)

Supplemental [Table 1. Modified CFIR-ERIC Mapping tool with CFIR constructs and selected strategies included in the multi-level HFrEF implementation bundle 0](#_Toc202822259)

Supplemental [Figure 1: Conceptual model illustrating interactions identified for the theme “Developing competency in HFrEF Care” 3](#_Toc202822260)

# Supplemental Methods. Beliefs about medicines questionnaire

Belief about medicines questionnaire – General

- I would like to ask you about your personal views about medicines in general.
- These are statements other people have made about medicines in general.
- Please indicate the extent to which you agree or disagree with them by ticking the
- appropriate box.
- There are no right or wrong answers. I am interested in your personal views.
- Please only tick one box per question.

*Rated on a Likert scale from (1=strongly disagree to 5=strongly agree)*

1. Doctors use too many medicines *(General-Overuse)*
2. People who take medicines should stop their treatment for a while every now and again. *(General-Harm)*
3. Most medicines are addictive. *(General-Harm)*
4. Natural remedies are safer than medicines. *(General-Overuse)*
5. Medicines do more harm than good. *(General-Harm)*
6. All medicines are poisons. *(General-Harm)*
7. Doctors place too much trust on medicines. *(General-Overuse)*
8. If doctors had more time with patients they would prescribe fewer medicines. *(General-Overuse)*

Belief about medicines questionnaire – Specific

- I would like to ask you about your personal views about medicines prescribed for your heart failure.
- These are statements other people have made about their heart failure medication.
- Please indicate the extent to which you agree or disagree with them by placing a cross in the appropriate box.
- There are no right or wrong answers. I am interested in your personal views.
- Please only cross one box per question

*Rated on a Likert scale from (1=strongly disagree to 5=strongly agree)*

1. My health at present depends on my heart failure medicines. *(Specific-Necessity)*
2. Having to take heart failure medication worries me. *(Specific-Concerns)*
3. My life would be impossible without my heart failure medication. *(Specific-Necessity)*
4. Without my heart failure medication I would be very ill. *(Specific-Necessity)*
5. I sometimes worry about the long-term effects of my heart failure medication. *(Specific-Concerns)*
6. My heart failure medication is mystery to me. *(Specific-Concerns)*
7. My health in the future will depend on my heart failure medication. *(Specific-Necessity)*
8. My heart failure medication disrupts my life. *(Specific-Concerns)*
9. I sometimes worry about becoming too dependent on my heart failure medication. *(Specific-Concerns)*
10. My heart failure medication protects me from becoming worse. *(Specific-Necessity)*

# Supplemental Results. Thematic analysis

**Interactions**

- Organizing Theme: Current state of HFrEF care – determinants affecting HFrEF care also affect a polypill-based strategy for HFrEF
- Temporal interaction:
  1. Awareness: What is the innovation and do I know it exists?
  2. Innovation acceptability: Does the innovation work?
  3. Innovation appropriateness: Would the innovation work for my practice?
  4. Implementation: What is my experience using the innovation?
  5. Decision: Should I continue to use the innovation?
- Developing competency in HFrEF care: (**Figure S2**)

**Theme 1: Awareness of new innovations**

- Delayed uptake due to lack of awareness
  - Dissemination strategies for HFrEF care
  - Dissemination strategies for HFrEF polypill
- Innovation acceptability
  - Supporting evidence required
    - Study design
    - Clinical outcomes
    - Implementation outcomes
    - Service outcomes
  - Trust in new innovations
    - People are more willing to change if the source is deemed trustworthy:
    - Behaviors of patients who trusted their doctor
    - Behaviors of patients who didn’t trust their doctor
    - Health literacy affects trust
    - Trusted sources for polypill approval
    - Untrustworthy sources for polypill approval

**Theme 2: Assessing innovation appropriateness**

- Patient Biopsychosocial characteristics influence provider practices
  - Clinical characteristics that affect HFrEF management
  - Psychosocial characteristics that affect HFrEF management
  - Polypill patient selection bias due to clinical characteristics
  - Polypill patient selection bias due to psychosocial characteristics
- Time during clinical encounters
- Support from non-physician providers/Access to non-physician providers to support polypill implementation
- Access to cardiovascular specialists/Access to physicians who prescribe the polypill
- System tools are not optimally implemented to support HFrEF care
  - EMR capabilities
  - Implementation strategies for improving HFrEF care
  - Implementation strategies for improving HFrEF polypill
- Institutional Constraints
  - Policies
  - Healthcare settings (e.g. admission, IPR, SNF) allow for monitoring while titrating medications
  - Titrating GDMT while monitoring for side effects
  - Monitoring while on the polypill
  - Access to pharmacies
- Costs of Care affects HFrEF management
  - Cost of medication affects treatment regimen
  - Non-Medication Costs
  - Costs limits accessibility to HFrEF polypill

**Theme 3: Developing competency in HFrEF Care**

- Treatment adherence
  - Readiness for change
  - Motivation for adherence
  - Patients who are more engaged are more able to manage their HFrEF
  - Patients modify their HFrEF regimen to observe effects
  - Observable effects affect prescribing practice
- Care coordination
  - Transitions of Care
  - Initiating and titrating GDMT
  - Titrating specific medications (BB, RAASi, MRA)
  - Large variety in HFrEF practice patterns
  - Large variation on proposed polypill practice
  - Coordinating care between healthcare providers
  - Managing adverse events with polypill
  - Care coordination required for the HFrEF polypill
- Therapeutic Inertia
  - Don’t prescribe all four HFrEF GDMT
  - Reluctant to uptitrate/eager to downtitrate HFrEF GDMT
  - Changing practice to incorporate new best practices
  - Delays in optimizing GDMT leads to worst patient outcomes
  - Patients are reluctant to modify their GDMT regimen
  - Providers defer to their cardiologist to manage HF medications
  - Polypill will not change the provider’s HFrEF practice

**Themes for strategy selection and mechanism mapping**

1. Strategies to support the HFrEF polypill
   1. Dissemination strategies: Strategies that increase polypill awareness and/or knowledge of how to manage polypill
      1. Provider education
      2. Patient education
      3. Education strategy
      4. Marketing/advertising
   2. Implementation Strategies
      1. Facilitation
      2. Adaptive and iterative strategies
         1. Staged implementation plan
         2. Adapting and tailoring strategies
         3. Pilot populations
         4. Vulnerable populations
      3. Tools
         1. EMR based strategies
         2. Other Quality management tools
      4. Simplify processes
      5. Available resources
         1. Utilizing non-physician healthcare providers
         2. Home based titration/monitoring
         3. Healthcare setting
      6. Reducing cost
   3. Stakeholders
      1. Pharmacy
      2. Champions (early adopters vs laggards)
      3. Non-physician providers
      4. Committees
      5. Physicians
      6. Patients
      7. Leadership
2. HFrEF polypill
   1. Innovation characteristics
      1. Polypill design
      2. Polypill safety
      3. Polypill adaptability
      4. Large variations in proposed polypill practice
   2. Managing adverse events with polypill
   3. Monitoring while on the polypill
   4. Care coordination for the HFrEF polypill
   5. Disparities with HFrEF polypill
      1. Polypill components are not optimal GDMT
      2. Cost limits accessibility to the polypill
      3. Threats to polypill access
      4. Selection bias due to psychosocial characteristics
      5. Selection bias due to clinical characteristics

#

# Supplemental Table 1. Modified CFIR-ERIC Mapping tool with CFIR constructs and selected strategies included in the multi-level HFrEF implementation bundle

| **CFIR Construct:** | Innovation Relative Advantage | Innovation Adaptability | Innovation Complexity | Innovation Cost | Partnerships & Connections | Culture | Implementation Climate | Tension for Change | Available Resources | Access to knowledge & information | Engaging: Deliverers | Engaging: Recipients | Executing | Count of ERIC Strategies | Count of Level 1 Strategies | Count of Level 2 Strategies |
| --- | --- | --- | --- | --- | --- | --- | --- | --- | --- | --- | --- | --- | --- | --- | --- | --- |
| **ERIC Strategies** |  |  |  |  |  |  |  |  |  |  |  |  |  |  |  |  |
| Assess for readiness and identify barriers and facilitators | 24% | 31% | 30% | 16% | 15% | 41% | **52%** | 35% | 13% | 7% | 38% | 14% | 31% | 14 | 1 | 8 |
| Conduct cyclical small tests of change | 31% | 23% | 37% | 8% | 0% | 0% | 11% | 4% | 13% | 3% | 8% | 9% | 7% | 12 | 0 | 4 |
| Conduct local needs assessment | 34% | 35% | 3% | 4% | 12% | 22% | 26% | 43% | 0% | 3% | 21% | 18% | 3% | 13 | 0 | 6 |
| Develop a formal implementation blueprint | 7% | 8% | 43% | 8% | 4% | 7% | 7% | 13% | 4% | 14% | 8% | 5% | 28% | 14 | 0 | 2 |
| Develop and implement tools for quality monitoring | 7% | 0% | 7% | 0% | 0% | 4% | 4% | 9% | 0% | 0% | 4% | 0% | 31% | 8 | 0 | 1 |
| Develop and organize quality monitoring systems | 3% | 4% | 10% | 4% | 0% | 0% | 7% | 9% | 0% | 0% | 8% | 5% | 21% | 10 | 0 | 1 |
| Obtain and use patients/consumers and family feedback | 7% | 4% | 0% | 4% | 0% | 7% | 7% | 9% | 0% | 0% | 13% | 41% | 0% | 9 | 0 | 1 |
| Purposely reexamine the implementation | 7% | 12% | 17% | 0% | 4% | 7% | 4% | 4% | 4% | 0% | 8% | 0% | 45% | 11 | 0 | 1 |
| Stage implementation scale up | 10% | 0% | 30% | 8% | 0% | 11% | 4% | 4% | 13% | 3% | 0% | 0% | 7% | 10 | 0 | 2 |
| Facilitation | 10% | 27% | 20% | 8% | 12% | 30% | 22% | 0% | 4% | 10% | 17% | 5% | 24% | 13 | 0 | 6 |
| Provide local technical assistance | 0% | 4% | 17% | 4% | 4% | 0% | 0% | 0% | 0% | 24% | 4% | 0% | 31% | 8 | 0 | 2 |
| Promote adaptability | 24% | **73%** | 40% | 16% | 0% | 22% | 15% | 17% | 4% | 7% | 17% | 5% | 10% | 13 | 1 | 4 |
| Tailor strategies | 17% | 35% | 27% | 12% | 0% | 30% | 19% | 13% | 9% | 0% | 17% | 9% | 10% | 12 | 0 | 4 |
| Build a coalition | 14% | 15% | 0% | 4% | **62%** | 19% | 19% | 9% | 17% | 3% | 25% | 18% | 0% | 12 | 1 | 1 |
| Capture and share local knowledge | 17% | 35% | 27% | 4% | 23% | 22% | 15% | 13% | 22% | 31% | 13% | 5% | 14% | 14 | 0 | 7 |
| Conduct local consensus discussions | 24% | 31% | 7% | 4% | 15% | 22% | 19% | 43% | 0% | 10% | 42% | 18% | 7% | 13 | 0 | 5 |
| Develop academic partnerships | 0% | 0% | 0% | 4% | **50%** | 0% | 4% | 4% | 4% | 10% | 8% | 0% | 0% | 8 | 1 | 0 |
| Identify and prepare champions | 45% | 23% | 30% | 12% | 15% | **52%** | 37% | 48% | 4% | 24% | **63%** | 23% | 14% | 14 | 2 | 7 |
| Identify early adopters | 17% | 27% | 20% | 8% | 4% | 11% | 30% | 13% | 0% | 10% | 13% | 9% | 14% | 13 | 0 | 3 |
| Inform local opinion leaders | 28% | 15% | 13% | 12% | 15% | 22% | 7% | 39% | 0% | 7% | 29% | 5% | 3% | 13 | 0 | 5 |
| Involve executive boards | 3% | 0% | 0% | 20% | 23% | 19% | 11% | 13% | 17% | 0% | 25% | 0% | 7% | 10 | 0 | 3 |
| Model and simulate change | 10% | 19% | 27% | 20% | 8% | 7% | 19% | 13% | 0% | 7% | 8% | 5% | 14% | 13 | 0 | 3 |
| **CFIR Construct:** | Innovation Relative Advantage | Innovation Adaptability | Innovation Complexity | Innovation Cost | Partnerships & Connections | Culture | Implementation Climate | Tension for Change | Available Resources | Access to knowledge & information | Engaging: Deliverers | Engaging: Recipients | Executing | Count of ERIC Strategies | Count of Level 1 Strategies | Count of Level 2 Strategies |
| **ERIC Strategies** |  |  |  |  |  |  |  |  |  |  |  |  |  |  |  |  |
| Organize clinician implementation team meetings | 14% | 8% | 20% | 0% | 0% | 4% | 11% | 9% | 9% | 14% | 4% | 0% | 24% | 11 | 0 | 2 |
| Promote network weaving | 3% | 4% | 0% | 0% | **50%** | 11% | 7% | 4% | 9% | 10% | 13% | 14% | 0% | 10 | 1 | 0 |
| Recruit, designate and train for leadership | 3% | 0% | 7% | 4% | 15% | 33% | 26% | 4% | 4% | 3% | 13% | 9% | 7% | 12 | 0 | 2 |
| Use advisory boards and workgroups | 10% | 4% | 0% | 0% | 35% | 22% | 11% | 13% | 4% | 0% | 25% | 32% | 10% | 11 | 0 | 4 |
| Use an implementation adviser | 7% | 8% | 10% | 4% | 8% | 4% | 7% | 13% | 13% | 14% | 4% | 9% | 21% | 14 | 0 | 2 |
| Visit other sites | 21% | 19% | 3% | 16% | 38% | 11% | 15% | 13% | 9% | 14% | 4% | 0% | 17% | 13 | 0 | 2 |
| Conduct educational meetings | 24% | 12% | 13% | 12% | 12% | 22% | 15% | 17% | 0% | **79%** | 21% | 18% | 3% | 13 | 1 | 3 |
| Conduct educational outreach visits | 10% | 12% | 7% | 4% | 23% | 4% | 7% | 4% | 0% | 28% | 13% | 9% | 14% | 13 | 0 | 2 |
| Conduct ongoing training | 3% | 0% | 37% | 0% | 0% | 4% | 11% | 0% | 9% | 38% | 4% | 0% | 28% | 9 | 0 | 3 |
| Create a learning collaborative | 7% | 23% | 33% | 8% | 31% | 30% | 19% | 9% | 9% | 45% | 33% | 5% | 21% | 14 | 0 | 7 |
| Develop educational materials | 14% | 12% | 13% | 0% | 4% | 0% | 0% | 0% | 4% | **59%** | 4% | 27% | 7% | 9 | 1 | 1 |
| Distribute educational materials | 10% | 12% | 3% | 0% | 0% | 4% | 0% | 13% | 0% | **55%** | 4% | 18% | 7% | 10 | 1 | 0 |
| Provide ongoing consultation | 3% | 8% | 20% | 0% | 0% | 15% | 15% | 4% | 0% | 17% | 8% | 0% | 24% | 10 | 0 | 2 |
| Shadow other experts | 3% | 12% | 7% | 0% | 4% | 7% | 4% | 4% | 0% | 21% | 0% | 0% | 0% | 9 | 0 | 1 |
| Develop resource sharing agreements | 7% | 0% | 0% | 32% | 31% | 0% | 0% | 0% | 26% | 3% | 4% | 5% | 0% | 8 | 0 | 3 |
| Facilitate relay of clinical data to providers | 10% | 4% | 3% | 0% | 0% | 4% | 7% | 22% | 0% | 10% | 4% | 5% | 10% | 11 | 0 | 1 |
| Increase demand | 24% | 4% | 3% | 12% | 0% | 4% | 7% | 13% | 4% | 0% | 8% | 9% | 0% | 10 | 0 | 1 |
| Intervene with patients/consumers to enhance uptake & adherence | 7% | 8% | 3% | 4% | 0% | 4% | 4% | 0% | 0% | 0% | 4% | **50%** | 3% | 9 | 1 | 0 |
| Involve patients/consumers and family members | 3% | 8% | 0% | 0% | 4% | 11% | 15% | 22% | 0% | 3% | 13% | **59%** | 3% | 10 | 1 | 1 |
| Prepare patients/consumers to be active participants | 0% | 0% | 0% | 0% | 0% | 7% | 7% | 9% | 0% | 0% | 13% | **55%** | 0% | 5 | 1 | 0 |
| Use mass media | 0% | 0% | 0% | 4% | 8% | 0% | 4% | 4% | 0% | 3% | 13% | 41% | 0% | 7 | 0 | 1 |
| Access new funding | 10% | 0% | 3% | **72%** | 4% | 0% | 0% | 0% | **78%** | 0% | 4% | 5% | 3% | 9 | 2 | 0 |
| Alter incentive/allowance structures | 28% | 0% | 7% | 44% | 0% | 15% | 44% | 22% | 17% | 0% | 17% | 0% | 17% | 9 | 0 | 4 |
| Alter patient/consumer fees | 0% | 0% | 0% | 20% | 0% | 0% | 4% | 0% | 22% | 0% | 0% | 23% | 0% | 4 | 0 | 3 |
| **CFIR Construct:** | Innovation Relative Advantage | Innovation Adaptability | Innovation Complexity | Innovation Cost | Partnerships & Connections | Culture | Implementation Climate | Tension for Change | Available Resources | Access to knowledge & information | Engaging: Deliverers | Engaging: Recipients | Executing | Count of ERIC Strategies | Count of Level 1 Strategies | Count of Level 2 Strategies |
| **ERIC Strategies** |  |  |  |  |  |  |  |  |  |  |  |  |  |  |  |  |
| Fund and contract for clinical innovation | 14% | 0% | 3% | 28% | 0% | 0% | 7% | 9% | 39% | 3% | 8% | 0% | 3% | 10 | 0 | 2 |
| Make billing easier | 0% | 4% | 3% | 32% | 0% | 0% | 4% | 0% | 22% | 0% | 0% | 0% | 3% | 6 | 0 | 2 |
| Place innovation on fee for service lists/formularies | 3% | 0% | 0% | 24% | 0% | 0% | 0% | 4% | 17% | 0% | 0% | 0% | 0% | 4 | 0 | 1 |
| Use other payment schemes | 0% | 0% | 0% | 20% | 0% | 0% | 0% | 0% | 22% | 0% | 0% | 0% | 0% | 2 | 0 | 2 |
| Change physical structure and equipment | 3% | 0% | 3% | 4% | 0% | 0% | 4% | 0% | 48% | 0% | 0% | 0% | 3% | 6 | 0 | 1 |
| **Count of ERIC Strategies** | **44** | **35** | **38** | **38** | **29** | **37** | **44** | **40** | **32** | **34** | **44** | **34** | **39** |  | **15** | **129** |

CFIR = Consolidated Framework for Implementation Research; ERIC = Expert Recommendations for Implementing Change

# Supplemental Figure 1: Conceptual model illustrating interactions identified for the theme “Developing competency in HFrEF Care”

HFrEF Symptoms

HFrEF status

Patient Treatment Adherence

Healthcare Provider HFrEF Guideline/Innovation Adherence
